# Sarcopenia Predicts Major Complications after Resection for Primary Hepatocellular Carcinoma in Compensated Cirrhosis

**DOI:** 10.3390/cancers14081935

**Published:** 2022-04-12

**Authors:** Giovanni Marasco, Elton Dajti, Matteo Serenari, Luigina Vanessa Alemanni, Federico Ravaioli, Matteo Ravaioli, Amanda Vestito, Giulio Vara, Davide Festi, Rita Golfieri, Matteo Cescon, Matteo Renzulli, Antonio Colecchia

**Affiliations:** 1 Internal Medicine and Digestive Pathophysiology Unit, IRCCS Azienda Ospedaliero-Universitaria di Bologna, 40138 Bologna, Italy; 2 Department of Medical and Surgical Sciences, University of Bologna, 40138 Bologna, Italy; e_dajti17@hotmail.com (E.D.); matteo.serenari@gmail.com (M.S.); vanessaalemanni1@gmail.com (L.V.A.); f.ravaioli@unibo.it (F.R.); matteo.ravaioli6@unibo.it (M.R.); daivde.festi@unibo.it (D.F.); matteo.cescon@unibo.it (M.C.); 3 Gastroenterology Unit, IRCCS Azienda Ospedaliero-Universitaria di Bologna, 40138 Bologna, Italy; amanda.vestito@aosp.bo.it; 4 General Surgery Division, IRCCS Azienda Ospedaliero-Universitaria di Bologna, 40138 Bologna, Italy; 5 Radiology Division, IRCCS Azienda Ospedaliero-Universitaria di Bologna, 40138 Bologna, Italy; giulio.vara@studio.unibo.it (G.V.); rita.golfieri@unibo.it (R.G.); dr.matteo.renzulli@gmail.com (M.R.); 6 Gastroenterology Unit, University Hospital of Modena, 41125 Modena, Italy; antonio.colecchia@unimore.it

**Keywords:** post-hepatectomy liver failure, liver resection, hepatocellular carcinoma, sarcopenia

## Abstract

**Simple Summary:**

Sarcopenia, which is defined as a loss of skeletal muscle mass, function and strength, is the result of major metabolic changes often observed in advanced liver disease. Its evaluation mirrors the nutritional and functional status of the patients, and thus has been recently implicated as an outcome predictor of patients with liver diseases and hepatocellular carcinoma. This study provides evidence that sarcopenia, as assessed by the skeletal muscle index, is associated with age and body mass index in liver surgery candidates. More importantly, it is associated with higher rates of major complications (Clavien-Dindo grade III or IV) in patients with compensated advanced chronic liver disease and/or portal hypertension undergoing liver resection for primary hepatocellular carcinoma.

**Abstract:**

The burden of post-operative complications of patients undergoing liver resection for hepatocellular carcinoma (HCC) is a cause of morbidity and mortality. Recently, sarcopenia has been reported to influence the outcome of patients with cirrhosis. We aimed to assess factors associated with sarcopenia and its prognostic role in liver surgery candidates. We included all patients with compensated advanced chronic liver disease (cACLD) undergoing liver resection for primary HCC consecutively referred to the University of Bologna from 2014 to 2019 with an available preoperative abdominal CT-scan performed within the previous three months. A total of 159 patients were included. The median age was 68 years, and 80.5% of the patients were male. Sarcopenia was present in 82 patients (51.6%). Age and body mass index (BMI) were associated with the presence of sarcopenia at multivariate analysis. Thirteen (8.2%) patients developed major complications and 14 (8.9%) presented PHLF grade B-C. The model for end-stage liver disease score was associated with the development of major complications, whereas cACLD presence, thrombocytopenia, portal hypertension (PH), Child-Pugh score and Albumin-Bilirubin score were found to be predictors of clinically significative PHLF. The rate of major complications was 11.8% in sarcopenic patients with cACLD compared with no complications (0%) in patients without sarcopenia and cACLD (*p* = 0.032). The rate of major complications was significantly higher in patients with (16.3%) vs. patients without (0%) sarcopenia (*p* = 0.012) in patients with PH. In conclusion, sarcopenia, which is associated with age and BMI, may improve the risk stratification of post-hepatectomy major complications in patients with cACLD and PH.

## 1. Introduction

Hepatocellular carcinoma (HCC) represents the fifth cancer worldwide, being diagnosed in compensated Advanced Chronic Liver Disease (cACLD) patients with an annual rate of 1–8% [1,2]. Patients with compensated liver disease including compensated Child-Pugh class A liver function with model for end-stage liver disease (MELD) score < 10, without portal hypertension (PH) and with an acceptable remaining parenchyma can be candidates for liver resection, which represents the best curative option for HCC [1,3,4]. Despite the latest improvements in clinical management and surgical techniques, the combination of the previous factors led to an expected post-hepatectomy liver failure (PHLF) incidence < 5% [1]. Indeed, PHLF contributes to a perioperative mortality < 3% and morbidity < 20%, while severe forms lead to death in up to 23% of cases [5]. As matter of fact, PHLF is closely related both to the resection extent and the background liver disease [6]. A careful assessment of pre-operative liver function and portal hypertension is required to recognize the best candidates for liver resection [3,7,8,9]. The Child-Pugh score, MELD score, and the evidence of clinical signs of PH or through the measurement of hepatic venous pressure gradient (HVPG) or the addition of an indocyanine green clearance [10] test are the most used pre-operative tools. However, their introduction in clinical practice appeared to be insufficient for predicting this complication. Consequently, many efforts have been made in the last decade to improve the PHLF risk stratification [6,11]. We previously found that an elevated liver and spleen stiffness measurement (LSM), which are also surrogate markers of PH [12,13,14,15], were associated with PHLF [4,7,16]. However, the available tools for PHLF prediction do not assess the general, nutritional, and functional status of HCC patients. A recent field of interest in hepatology is the evaluation of the nutritional and functional status of patients mirrored by the assessment of sarcopenia [17,18]. Sarcopenia, described as a loss of skeletal muscle mass, function and strength, is the result of major metabolic changes often observed not only in oncologic settings or in the elderly, but even in advanced liver disease [17]. As an example, sarcopenia has been reported in 22–65% of cirrhotic patients and is associated with a twofold higher risk of waiting list mortality and a worse outcome after OLT [19]. The most validated tools for sarcopenia assessment are the measurement of skeletal muscle areas (SMA, cm2) or the index (SMI) using cross sectional images acquired by computed tomography (CT) or magnetic resonance imaging (MRI), such us at the level of psoas muscles or the third lumbar vertebra (L3) [18]. To our knowledge, only a Japanese study [20] evaluated the impact of total psoas muscle area (TPA) as a sarcopenia surrogate for predicting PHLF and other post-hepatectomy complications. However, data from Western countries using the more precise SMA or SMI assessment for sarcopenia are still lacking. Thus, we aimed to assess factors associated with sarcopenia in liver surgery candidates and the prognostic role of sarcopenia evaluated with SMI for predicting the development of major complications (Clavien-Dindo classification III or IV) or liver failure (clinically significative PHLF) in a 10-year cohort of consecutively enrolled patients undergoing liver resection for HCC in a European third-level referral center. 

## 2. Materials and Methods

### 2.1. Patients Selection and Data Collection

All patients undergoing liver resection for primary HCC consecutively enrolled at the Department of Medical and Surgical Sciences of the University of Bologna, Bologna, Italy from November 2014 to December 2019 were included in this study. Patients with an available preoperative abdominal CT-scan performed within three months prior to surgery were finally included in the study. We excluded patients undergoing other intra-operative treatments, such us radiofrequency ablation, or that had other malignant tumors or metastatic disease and those without preoperative CT images (e.g., patients with CT-scan performed in other centers or with magnetic resonance imaging) or incomplete medical records. After enrollment, demographic and clinical data from medical records were collected, including pre-operative laboratory tests, LSM measurements, tumor-related characteristics, sarcopenia assessed on an abdominal CT-scan, 30-days post-operative complications, and mortality. The types and extent of liver resection were also registered. The study was conducted following the Helsinki Declaration and approved by the local Ethical Committee. 

### 2.2. Sarcopenia Assessment

For sarcopenia assessment, two experienced radiologists revised all abdominal CT-images for SMI assessment. Briefly, following the description by Gomez-Perez et al. [21] for the use of the free public domain software developed by the National Institutes of Health (NIH ImageJ): starting from a single cross-sectional image at the midpoint of L3, a manual tracing of abdominal regions was performed to delineate body composition. After tracing the abdominal perimeter as a surrogate for the waist circumference, the operator draws the abdominal muscles inner and outer perimeters. Using a pre-programmed template skeletal muscle area was thus determined and then combined with the squared height of the patient, finally generating the SMI (cm^2^/m^2^).

### 2.3. Definitions

Sarcopenia was defined according to the definition endorsed by the European Association for the Study of the Liver as SMI < 50 cm^2^/m^2^ in male patients and SMI < 39 cm^2^/m^2^ in female patients [22,23]. LSM was assessed by the Transient Elastography technique with Fibroscan (Echosens, Paris, France), performed on the right hepatic lobe after an ultrasound (US) preliminary view. Reliable LSM assessments were established as previously reported [24], with at least ten valid measurements, an IQR to median value ratio < 0.3 and a success rate >60%; cACLD was defined by LSM ≥ 10 kPa. The ALBI score is a new non-invasive tool to evaluate liver function, predict survival and post-hepatectomy complications [25,26]. It is calculated according to serum albumin and total bilirubin levels, calculated with the formula: (log10 bilirubin [µmol/L] × 0.66) + (albumin [g/L] × −0.0852). It was further categorized into three different grades: ALBI 1 (≤−2.60), ALBI 2 (>−2.60 to ≤−1.39), and ALBI 3 (>−1.39) [27]. Surgical complications were evaluated according to Clavien-Dindo classification [28], including any complications requiring surgical or radiologic intervention, life-threatening organ failure, and death. Major complications were defined for grade III or IV complications. Specifically, a PHLF diagnosis was performed according to the International Study Group of Liver Surgery (ISGLS) definition [29], in case of increased INR and concomitant hyperbilirubinemia after five postoperative days. The severity of PHLF was graded as follows: Grade A PHLF, requiring no specific treatment; grade B PHLF requiring essential non-invasive treatment (transfusion support, albumin supplementation, diuretic therapy); and grade C PHLF requiring invasive procedures, including mechanical ventilation, hemodialysis or extracorporeal liver support [29]. PHLF grade B and C were considered clinically significant [29]. 

### 2.4. Outcomes

The primary study outcome was the evaluation of factors associated with sarcopenia in liver surgery candidates; as a secondary outcome we aimed to assess the usefulness of sarcopenia evaluation for predicting the development of major complications (Clavien-Dindo classification III or IV) or liver failure (clinically significative PHLF). 

### 2.5. Statistical Analysis

Categorical data were expressed as numbers (percentages), and continuous variables as medians (interquartile range). For group comparisons of categorical and continuous variables, the chi-square test or Mann-Whitney test, and the McNemar test were used as required. The relationships between sarcopenia and other demographical and clinical factors were assessed using the Spearman’s Rho. Briefly, this test is used to measure the strength of association between two variables, where the value r = 1 means a perfect positive correlation and the value r = −1 means a perfect negative correlation. Comparisons and associations have been graphically translated in dot or scatter plots when appropriate. Factors associated with the presence of sarcopenia among liver surgery candidates were assessed with several univariate logistic regression analyses and, subsequently, with a stepwise multivariate logistic regression approach. Also, the association between sarcopenia and the development of major complications and clinically significative PHLF was evaluated by logistic regression analysis. Subgroup analyses were performed according to these pre-specified categories: age (<65 vs. ≥65 years old), gender (male vs. female), ACLD presence (LSM < 10 kPa vs. ≥10 kPa), presence of portal hypertension (defined by either esophageal varices, PLT < 150 or LSM > 20 kPa), liver function (ALBI grade 1 vs. ALBI grade 2 or 3), type of surgery (minor hepatectomy vs. major hepatectomy). All *p* values referred to two-tailed tests of significance. *p* < 0.05 was considered significant. The statistical analysis was carried out using Stata/SE (Version 14.0; Stata Corp, College Station, TX, USA).

## 3. Results

### 3.1. Patients’ Characteristics

**Figure 1:**
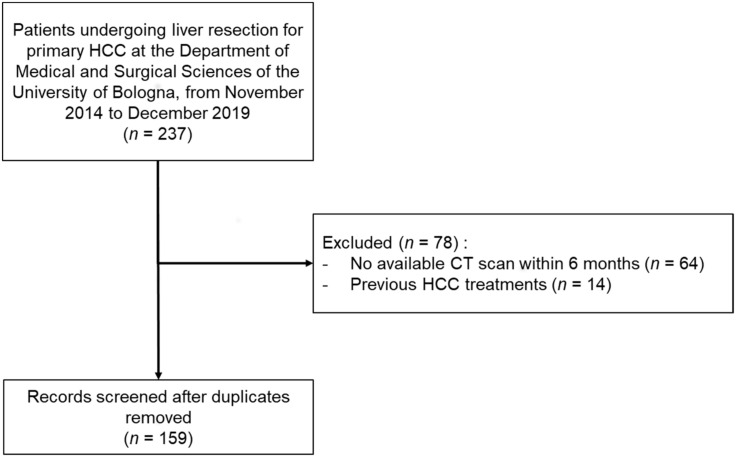
Flow-chart for the enrollment in the study.

Two-hundred and thirty-seven patients underwent liver resection for primary HCC at the Department of Medical and Surgical Sciences of the University of Bologna within the study period. After excluding 78 patients (Figure 1), a total of 159 patients were included in the final analysis. The median age was 68 (58–75) years; patients were mostly male (128, 80.5%), overweight (69.2%), and with viral etiology of liver disease (106, 66.7%). Regarding liver severity, median LSM was 16.4 (10.1–25.4) kPa, thrombocytopenia and portal hypertension were present in 45.9% and 57.9%, respectively, median MELD was 8 (7–9). Median HCC size was 37 mm, (23–60) and 40 (25.2%) patients underwent a major hepatectomy. Median SMI was 39.2 (35.7–42.4) cm^2^/m^2^ and 48.9 (43–55.3) cm^2^/m^2^ in female and male patients, respectively. Overall sarcopenia was present in 82 patients (51.6%). Patients with and without sarcopenia differ significantly in age and BMI, but not in liver disease severity or HCC characteristics. Demographics, clinical and tumor-related data are reported in Table 1.

### 3.2. Factors Associated with the Presence of Sarcopenia among Liver Surgery Candidates

**Figure 2:**
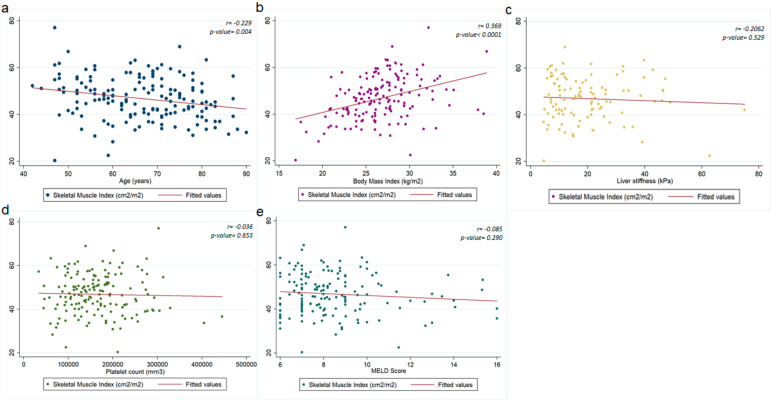
Correlation between sarcopenia by skeletal muscle index (SMI) and: (**a**) age, (**b**) body mass index (BMI), (**c**) liver stiffness measurement (LSM), (**d**) platelet count and (**e**) Model for end-stage liver disease (MELD). r = Spearman’s Rho.

In patients with HCC undergoing hepatic resection, SMI values correlated significantly with age (r = −0.229, *p*-value = 0.004) and BMI (r = 0.369, *p*-value < 0.0001), but not with the severity of liver disease, as evaluated by liver stiffness (*p* = 0.529), platelet count (0.653) or MELD score (0.290) (Figure 2). In univariate analysis, age and BMI were the only two variables associated with the presence of sarcopenia. This association remained significant also in multivariate analysis, with OR of 1.045 (95%-CI:1.015–1.078) for age and 0.879 (95%-CI:0.802–0.962) for BMI (Table 2).

### 3.3. Factors Associated with Complications after Hepatic Resection 

**Figure 3:**
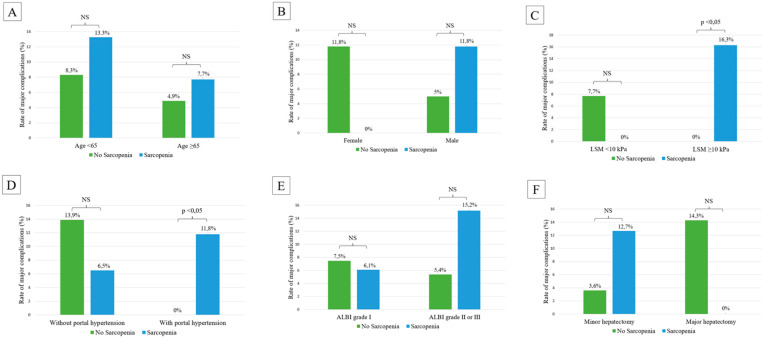
Rate of major complications according to the following sub-groups analysis: (**A**) age, (**B**) gender, (**C**) liver stiffness measurement (LSM), (**D**) portal hypertension, (**E**) albumin-bilirubin (ALBI) grade, (**F**) extent of hepatectomy.

During follow-up, 13 (8.2%) patients developed major complications and 48 (30.2%) PHLF, of whom 14 (8.9%) presented grade B or C PHLF. Factors associated with the development of major complications and clinically significant PHLF are reported in Table 3. Only the MELD score was associated with the development of major complications, whereas the ACLD presence defined as LSM ≥ 10 kPa, thrombocytopenia, portal hypertension, Child-Pugh score and ALBI score were found to be predictors of clinically significative PHLF. In the overall cohort, the presence of sarcopenia, evaluated both as a dichotomic variable (according to EASL recommendation [23]) and continuous variable (SMI in cm^2^/m^2^), was not significantly associated with the development of major complications (9.8% in presence vs. 6.5% in absence of sarcopenia), with an OR:1.557, 95%-CI: 0.486–4.983 and OR:0.975, 95%-CI: 0.918–1.036, respectively. Similarly, no association was found between the presence of sarcopenia and the presence of clinically significant PHLF (11% in presence vs. 6.6% in absence of sarcopenia), with an OR of 1.751 (95%-CI: 0.559–0.547) and 0.972 (95%-CI: 0.917–1.032), respectively. However, in the subgroup analyses, SMI as a continuous variable was significantly and inversely associated with the risk of major complications (OR: 0.889, 95%-CI: 0.798–0.990) in patients with portal hypertension. In fact, the rate of major complications was 11.8% in sarcopenic patients with portal hypertension compared with no complications (0%) in patients without sarcopenia and portal hypertension, and the difference was statistically significant (*p* = 0.032) (Figure 3). Similarly, in the subgroup of patients with cACLD, defined by LSM ≥10 kPa, the rate of major complications was significantly higher in patients with (16.3%) vs. patients without (0%) sarcopenia (*p* = 0.012). No other significant association was found between sarcopenia and major complications in the subgroups determined by age, gender, liver function, and type of surgery or between sarcopenia and clinically significant PHLF (Supplementary Table S1).

## 4. Discussion

This study provides evidence that sarcopenia, as assessed by the skeletal muscle index, is associated with age and BMI in liver surgery candidates. More importantly, it is associated with higher rates of major complications (Clavien-Dindo grade III or IV) in patients with cACLD and/or portal hypertension undergoing liver resection for primary hepatocellular carcinoma. 

The recent guidelines of the EASL currently suggest the inclusion of an assessment of sarcopenia within the nutritional assessment of cirrhotic patients [23]. Its relevance was first highlighted in the liver transplant waitlist setting [30,31,32], and then revealed even for the survival of cirrhotic patients independently of age, etiology and liver function. Moreover, several studies have found that sarcopenia was an independent risk factor for the prognosis of HCC in patients undergoing surgical resection [33] and other curative treatments [34,35,36]. However, there is a lack of knowledge on the effect of sarcopenia on short-term outcomes after resection, such as the occurrence of major complications or PHLF. We found that sarcopenia was very common among patients with HCC undergoing hepatic resection, as it was present in 82 out of the 159 (52%) of the included patients. In our cohort, SMI correlated well with age and BMI, but not with severity of liver disease (evaluated by LSM, platelet count or MELD score); in fact, only the former two variables were found to be independent factors associated with sarcopenia, as defined by EASL guidelines [23]. These results, even though expected, can help the clinicians quickly identify patients that are more likely to present sarcopenia (e.g., older patients with lower BMI), are at higher risk of morbidity and mortality, and could benefit from interventions aiming to improve pre-operative sarcopenia. The main result of our study was that the presence of sarcopenia was associated with a higher risk of developing major complications after hepatic resection in patients with advanced liver disease and portal hypertension. Among the included patients, 77% had cACLD (LSM ≥ 10 kPa) and only 58% presented signs of portal hypertension; however, this group represented the category of patients with more severe liver disease, where the establishment of sarcopenia synergically and negatively influenced patients’ prognosis, as reflected by the higher morbidity rate after hepatic resection, namely 12–16% versus 0% in patients without cACLD or portal hypertension.

To the best of our knowledge, only a Japanese study [20] has evaluated the impact of total psoas muscle area (TPA) for predicting PHLF, finding that patients with sarcopenia had a significantly higher rate of clinically significative PHLF, namely grade B and C (33% vs. 16%, *p* = 0.003), major complications assessed with Clavien grade C3 (54 vs. 37 %, *p* = 0.011), other than intra-abdominal abscess (29 vs. 18 %, *p* = 0.040), and longer hospitalization (39 vs. 30 days, *p* < 0.001) than those without sarcopenia. However, the etiology of the included patients was different, as most patients presented with biliary tract cancer undergoing major hepatectomy with extra-hepatic duct resection. Moreover, these authors [20] assessed sarcopenia using TPA [20] finding a best cut-off for development of liver failure for male of 567 mm^2^/m^2^, and for female of 395 mm^2^/m^2^, respectively (OR 2.44, 95% CI 1.20–4.99, *p* = 0.012). On the other hand, it has been previously reported that SMI is a more complete and robust measurement than psoas muscle-based sarcopenia measurements, especially in men with cirrhosis [37], since it includes a larger proportion of total muscle mass than other methods. However, it should be also underlined that the cut-off value for sarcopenia defined by the SMI has not been agreed upon [38]. Aside from mirroring the patient’s nutritional status, sarcopenia is also an indirect marker of patient frailty, defined as a syndrome characterized by a decreased physiological reserve which can influence the post-operative outcomes. Only one study reported a specific association between frailty and PHLF [39]. However, while the frailty assessment is based on a self-reporting questionnaire which could be affected by bias, sarcopenia can be objectively evaluated from CT- images using simple free software.

As mentioned above, patients with a single HCC nodule, a compensated cirrhosis as assessed by a Child-Pugh A class and normal bilirubin (<1 mg/dL), without portal hypertension, have the best prognosis and are ideal candidates for liver resection [3]. As a matter of fact, in Western countries the selection of candidates for resection is usually based on the assessment of portal hypertension, as clinically assessed (platelet count < 100,000/mL, associated with splenomegaly or esophageal varices) or by the measurement of the HVPG > 10 mmHg, which is an invasive and costly method [40]. The presence of clinically significant portal hypertension appears to be associated with a worse prognosis but does not preclude resection in selected patients [41,42]. However, even the role of portal hypertension assessed by clinical symptoms or HVPG has been recently debated, mainly due to the heterogeneity of endpoints and definitions used [43,44]. The heterogeneity regarding data for the usefulness of pre-operative assessment may often lead to different local strategies, which may not completely catch the patient general status or the degree of the underlying liver cirrhosis and HCC related characteristics, which may together contribute and be influenced by each other. Interestingly, previous studies showed no correlation between the degree of portal hypertension and sarcopenia [45,46], while keeping an independent prognostic role in cirrhotic patients [46]. This suggests that the two pathological processes are independent and should be both evaluated before liver resection to obtain a comprehensive patient risk stratification. This may be partially explained by the fact that the depletion of muscle mass in HCC patients seems to be dependent also on the aggressiveness of the HCC, which may lead to cachexia, rather than the underlying liver disease [19]. Interestingly, in line with these pathophysiological postulations, we found in our cohort that patients with a higher risk of post-operative complications were those with advanced cirrhosis, portal hypertension and sarcopenia. Our results open new frontiers, highlighting the utility of nutritional supplementation and physical exercise before liver resection, even more in patients with advanced cirrhosis and portal hypertension, similar to interventional studies performed before trans-arterial chemoembolization [47] and during permanence in the transplant waitlist [48,49]. 

Our study has some limits. First, due to the monocentric design, the sample size and the number of events (major complications and PHLF) may have lowered the statistical power of the study, thus influencing our results and preventing us from performing logistic multivariable analyses in some cases. However, the expected number of events is in line with that previously reported by other studies [6,42,50,51]. Another limiting factor is the retrospective design, which was partially overcome by including patients consecutively enrolled, thus minimizing the risk of a selection bias. Besides, the retrospective design may have also influenced the completeness of our data on LSM and the presence of esophageal varices. Moreover, being a tertiary referral center, a considerable rate of patients with comorbidities and with borderline indications for HCC treatment underwent surgery; this may have increased the rate of complications, biasing our results. Finally, since this was an exploratory pivotal study, we did not explore possible treatments for sarcopenia, such as physical exercise and nutritional supplementations [19].

## 5. Conclusions

In conclusion, the addition of sarcopenia evaluation with simple and free software may help clinicians and surgeons in the pre-operative assessment for hepatectomy for primary hepatocellular carcinoma. Thus, sarcopenia may improve the risk stratification of post-operative major complications in patients with advanced liver disease and portal hypertension. However, further studies are still needed to confirm this data and to evaluate the impact of peri-operative nutritional and rehabilitative intervention.

## Figures and Tables

**Table 1:** Characteristics of the patients included in the study.

Variables	All Patients (*n* = 159)	Patients with Sarcopenia (*n* = 82)	Patients without Sarcopenia (*n* = 77)	*p*-Value
Age	68 (58–75)	71 (60–78)	65 (54–71)	0.003
Sex (male)	128 (80.5%)	68 (82.93%)	60 (77.92%)	0.426
BMI (kg/m^2^)	26.3 (24.4–28.7)	25.6 (23.8–27.8)	27.5 (25.6–29.4)	0.0009
Overweight (BMI > 25 kg/m^2^)	110 (69.18%)	47 (57.32%)	63 (81.8%)	0.001
**Liver disease etiology**				0.385
Viral	106 (66.67%)	55 (67.07%)	51 (66.23%)	
NAFLD	24 (15.09%)	14 (17.07%)	10 (12.99%)	
ALD	6 (3.77%)	2 (2.44%)	4 (5.19%)	
Other	23 (14.47%)	11 (13.75%)	12 (15.58%)	
**Co-morbidities**				
Diabetes mellitus	46 (28.93%)	25 (30.49%)	21 (27.27%)	0.655
**Liver disease severity**				
LSM (kPa) (*n* = 108)	16.4 (10.1–25.4)	17.3 (10.1–26.3)	14 (10.1–25.1)	0.499
Platelets (cells × 10^9^/L)	158 (115–202)	148 (115–211)	165 (119–194)	0.658
Esophageal varices (*n* = 154)	44 (28.57%)	22 (27.16%)	22 (30.14%)	0.683
Portal hypertension	92 (57.86%)	51 (62.2%)	41 (53.25%)	0.253
MELD score	8 (7–9)	8 (7–10)	8 (7–9)	0.290
Child-Pugh score	5 (5–5)	5 (5–6)	5 (5–5)	0.275
ALBI grade > 1	70 (44.03%)	33 (40.24%)	37 (48.05%)	0.322
**Liver cancer**				
Alpha-fetoprotein	17 (6–103)	18 (4–94)	16 (7–125)	0.666
Number of HCC	1 (1–1)	1 (1–1)	1 (1–1)	0.923
Maximum diameter of HCCnodule (mm)	37 (23–60)	38 (25–55)	35 (23–60)	0.811
Major hepatectomy	40 (25.2%)	19 (23.17%)	21 (27.27%)	0.551

ALBI: albumin-bilirubin score; ALD: alcohol-related liver disease; BMI: body mass index; HCC: hepatocellular carcinoma, LSM: liver stiffness measurement; MELD: Model for end-stage-liver-disease; NAFLD: Non-alcoholic fatty liver disease.

**Table 2:** Factors associated with the presence of sarcopenia in liver surgery candidates.

Variables	Univariate Analysis		Multivariate Analysis	
	OR (95%-CI)	*p*-Value	OR (95%-CI)	*p*-Value
Age	1.046 (1.015–1.078)	0.003	1.046 (1.014–1.079)	0.004
Sex (male)	1.376 (0.626–3.026)	0.427		
BMI (kg/m^2^)	0.878 (0.802–0.962)	0.005	0.879 (0.801–0.965)	0.007
Diabetes mellitus	1.170 (0.588–2.326)	0.655		
LSM (kPa) (*n* = 108)	1.165 (0.476–2.850)	0.739		
Platelets (cells ×10^9^/L)	0.999 (0.999–1.0001)	0.726		
Esophageal varices (*n* = 154)	0.864 (0.429–1.740)	0.683		
Portal hypertension	1.445 (0.768–2.718)	0.254		
MELD score	1.089 (0.949–1.250)	0.225		
Child-Pugh score	1.215 (0.684–2.159)	0.506		
ALBI score	0.837 (0.550–1.273)	0.405		
Maximum diameter of HCC nodule (mm)	1.0001 (0.991–1.009)	0.988		
Number of HCC nodules	0.956 (0.614–1.289)	0.842		

ALBI: albumin-bilirubin score; BMI: body mass index; CI: confidence interval; HCC: hepatocellular carcinoma; LSM: liver stiffness measurement; MELD: Model for end-stage-liver-disease; OR: odds ratio.

**Table 3:** Factors associated with the development of major complications and PHLF B or C after hepatic resection for HCC.

	Major Complications		PHLF B or C	
Variables	Univariate Analysis		Univariate Analysis	
	OR (95%-CI)	*p*-Value	OR (95%-CI)	*p*-Value
Age	0.974 (0.926–1.026)	0.322	0.980 (0.933–1.030)	0.432
Sex (male)	1.363 (0.286–6.491)	0.697	0.577 (0.168–1.979)	0.382
BMI (kg/m^2^)	1.037 (0.895–1.201)	0.631	0.890 (0.760–1.041)	0.146
Diabetes mellitus	2.271 (0.720–7.170)	0.162	0.971 (0.289–3.271)	0.963
LSM > 10 kPa (*n* = 108)	2.660 (0.512–13.822)	0.244	13.565 (1.705–107.907)	0.014
Thrombocytopenia	1.011 (0.329–3.154)	0.985	4.849 (1.297–18.127)	0.019
Esophageal varices (*n* = 154)	0.429 (0.091–1.740)	0.284	2.757 (0.906–8.391)	0.074
Portal hypertension	0.598 (0.191–1.868)	0.376	3.384 (1.076–10.642)	0.037
MELD score	1.211 (1.018–1.442)	0.031	1.144 (0.962–1.360)	0.127
Child-Pugh score	2.050 (0.684–2.159)	0.085	2.218 (1.008–4.883)	0.048
ALBI score	1.981 (0.715–5.488)	0.188	7.195 (2.171–23.844)	0.001
Major hepatectomy	0.884 (0.231–3.385)	0.857	0.818 (0.216–3.097)	0.768
Sarcopenia (dichotomous)	1.557 (0.486–4.983)	0.453	1.751 (0.559–5.479)	0.336
SMI (continuous)	0.975 (0.918–1.036)	0.410	0.972 (0.917–1.032)	0.362

ALBI: albumin-bilirubin score; BMI: body mass index; CI: confidence interval; HCC: hepatocellular carcinoma; LSM: liver stiffness measurement; MELD: Model for end-stage-liver-disease; OR: odds ratio; PHLF: post-hepatectomy liver failure; SMI: skeletal muscle index.

## Data Availability

Data is contained within the article or Supplementary Material.

## Supplementary Materials

The following supporting information can be downloaded at: https://www.mdpi.com/article/10.3390/cancers14081935/s1, Table S1. Association between sarcopenia and complications in different subgroups.
