# Supplementary material for: Sarcopenia Predicts Major Complications after Resection for Primary Hepatocellular Carcinoma in Compensated Cirrhosis
Source: Cancers (Basel). 2022 Apr 12;14(8):1935. doi: 10.3390/cancers14081935 (PMC9025609; doi:10.3390/cancers14081935)
Supplement: Supplementary file 1 [file cancers-14-01935-s001.zip › cancers-1686362-supplementary.pdf]

cancers

MDPI

Supplementary Materials

Sarcopenia predicts major complications after resection for primary hepatocellular carcinoma in compensated cirrhosis.

Giovanni Marasco, Elton Dajti, Matteo Serenari, Luigina Vanessa Alemanni, Federico Ravaioli, Matteo Ravaioli, Amanda Vestito, Giulio Vara, Davide Festi, Rita Golfieri, Matteo Cescon, Matteo Renzulli and Antonio Colecchia

Table S1. Association between sarcopenia and complications in different subgroups.

	Outcome: Liver failure (PHLF B or C)					Outcome: Major complication				
	Sarcopenia presence				SMI (cm^2/m^2)	Sarcopenia presence				SMI (cm^2/m^2)
	**No**	**Yes**	**p-value**	**OR (95%-CI)**	**OR (95%-CI)**	**No**	**Yes**	**p-value**	**OR (95%-CI)**	**OR (95%-CI)**
**Age**										
<65 years (n=66)	3/36 (8.33%)	5/30 (16.7%)	0.302	2.200 (0480-10.087)	0.980 (0.908-1.053)	3/36 (8.3%)	4/30 (13.3%)	*0.511*	1.692 (0.348-8.238)	0.949 (0.877-1.026)
≥ 65 years (n=92)	2/40 (5%)	4/52 (7.7%)	0.604	1.583 (0.275-9.111)	0.954 (0.859-1.060)	2/41 (4.9%)	4/52 (7.7%)	*0.583*	1.625 (0.283-9.344)	1.002 (0.929-1.078)
**Gender**										
Female (n=31)	2/17 (11.8%)	2/14 (14.3%)	*0.835*	1.25 (0.153-10.226)	1.048 (0.867-1.266)	2/17 (11.8%)	0/14 (0%)	*0.185*	1 (omitted)	1.267 (0.915-1.754)
Male (n=128)	3/59 (5.1%)	7/68 (10.3%)	*0.277*	2.142 (0.017-0.171)	0.969 (0.012-11.965)	3/60 (5%)	8/68 (11.8%)	*0.173*	2.533 (0.640-10.025)	0.947 (0.881-1.017)
**Presence of advanced chronic liver disease (n=108)**										
LSM <10 kPa (n=25)	0	0	-	-	-	1/13 (7.7%)	0/12 (0%)	*0.327*	1 (omitted)	1.080 (0.831-1.403)
LSM ≥10 kPa (n=83)	5/40 (12.5%)	9/43 (20.9%)	0.305	1.852 (0.563-6.095)	0.967 (0.909-1.030)	0/40 (0%)	7/43 (16.3%)	0.012	1 (omitted)	*0.922 (0.842-1.010)*
**Portal hypertension presence**										
No portal hypertension (n=94)	2/46 (4.4%)	2/48 (4.2%)	0.965	0.965 (0.129-7.089)	0.987 (0.913-1.093)	5/36 (13.9%)	2/31 (6.5%)	*0.321*	0.428 (0.077-2.239)	1.016 (0.960-1.076)
Portal hypertension (n=64)	3/30 (10%)	7/34 (20.6%)	0.244	0.253 (0.545-9.986)	0.234 (0.046-57.258)	0/41 (0%)	6/51 (11.8%)	0.032	1 (omitted)	0.889 (0.798-0.990)
**Liver function**										
ALBI grade I (n=89)	1/39 (2.6%)	1/49 (2%)	0.870	0.792 (0.048-13.074)	1.073 (0.916-1.258)	3/40 (7.5%)	3/49 (6.1%)	0.797	0.804 (0.153-4.221)	0.998 (0.912-1.093)
ALBI grade II or III (n=70)	4/37 (10.8%)	8/33 (24.2%)	0.137	2.64 (0.714-9.764)	0.941 (0.874-1.012)	2/37 (5.4%)	5/33 (15.2%)	0.175	3.125 (0.563-17.338)	0.951 (0.871-1.038)
**Type of surgery**										
Minor hepatectomy (n=119)	5/56 (8.9%)	6/63 (52.9%)	0.911	1.073 (0.309-3.730)	0.967 (0.904-1.034)	2/56 (3.6%)	8/63 (12.7%)	*0.073*	3.927 (0.797-19.342)	0.957 (9.892-1.027)
Major hepatectomy (n=40)	0/20 (0%)	3/19 (15.8%)	0.064	1	0.990 (0.894-1.097)	3/21 (14.3%)	0/19 (0%)	*0.087*	1 (omitted)	1.010 (0.935-1.091)

ALBI: albumin-bilirubin score; CI: confidence interval; LSM: liver stiffness measurement; OR: odds ratio; SMI: skeletal muscle index
